# Improving data on homelessness and health: partnering with community-based organizations

**DOI:** 10.1186/s12889-024-20954-3

**Published:** 2025-08-26

**Authors:** Megan Schoonveld, Adam Hammond, Vanessa Li, Emily Mosites, Kristie E. N. Clarke

**Affiliations:** 1 Centers for Disease Control and Prevention, Office of the Deputy Director for Infectious Diseases, Atlanta, GA USA; 2 Oak Ridge Institute for Science and Education Fellowship, Oak Ridge Associated Universities, Oak Ridge, TN USA; 3 The MITRE Corporation, McLean, VA USA; 4 Centers for Disease Control and Prevention, Center for Surveillance, Epidemiology, and Laboratory Services, Atlanta, GA USA

**Keywords:** Technical expert panel, People experiencing homelessness, Community-based organizations

## Abstract

**Background:**

Community-based organizations (CBOs) provide critical services to people experiencing homelessness and played a unique role in data collection throughout the COVID-19 pandemic. Although data from CBOs filled a critical need, many jurisdictions faced challenges with timely and integrated data collection. We gathered expert opinions from CBO leaders on homelessness and health to identify how to support CBO data collection to best inform public health practices.

**Methods:**

We conducted purposively sampled semi-structured key informant interviews (KII) with CBO leaders. Questions included populations served, methods for collecting and sharing data, challenges during data collection and sharing, and possible solutions. KII transcripts were examined using thematic analysis. After the KIIs, we convened a technical expert panel (TEP) to review findings and suggest potential opportunities for improving data collection and sharing among CBOs.

**Results:**

We conducted 20 key informant interviews representing 16 CBOs. Three thematic areas emerged from the transcripts: challenges in data entry and collection, infrastructure limitations, and challenges to data sharing and partnerships. The 20 TEP members suggested that creating an interagency health and housing a data modernization support center could increase federal coordination, that system interoperability could be improved by creating standards for defining data elements and that more tools are needed to support CBOs to implement best practices.

**Discussion:**

TEP subject matter experts provided field-endorsed perspectives to support CBOs who work closely with people experiencing homelessness.

**Conclusion:**

Through increased collaboration at all levels and coordinated action, improved data to better support the health of people experiencing homelessness is an achievable goal.

**Supplementary Information:**

The online version contains supplementary material available at 10.1186/s12889-024-20954-3.

## Introduction

The COVID-19 pandemic brought the importance of data collection for responding to public health emergencies to the forefront, especially for people who are at increased risk for illness. People experiencing homelessness (PEH) are at increased risk of infectious disease [1], and more than half a million people in the United States experience homelessness on any single night [2]. However, data systems used by the healthcare system, public health professionals, and their partners often do not include indicators for housing status, which creates barriers in understanding the burden of disease in PEH [3]. Integrated data systems are needed to identify trends that can be used to inform the best prevention measures for infectious disease outbreaks among PEH [4].

Community-based organizations (CBOs) that provide homelessness services [5] are critical to supporting these communities, and they played a unique role during public health emergencies [6]. Their ability to collect data relevant to PEH makes them vital partners for public health decision makers seeking to effectively serve both emergent and ongoing needs of PEH. CBOs are in a unique position to collect data related to homelessness, as they fulfill the real-time service and support needs of PEH [7]. For example, the US Department of Housing and Urban Development (HUD) requires that HUD-funded CBOs collect person-level demographic data, including veteran and disability status, about those who are accessing homeless services in a Homeless Management Information System (HMIS) [8].

During the COVID-19 pandemic, national-level case data did not routinely include information on housing status and were therefore insufficient to assess the burden of COVID-19 among PEH [9]. The Centers for Disease Control and Prevention (CDC) began in-depth work to improve data collection on homelessness. The aim of this project was to identify recommendations for how to support CBOs in data collection and sharing for people experiencing homelessness.

## Methods

This project was conducted by the Health Federally Funded Research and Development Center, operated by the MITRE Corporation (hereafter referred to as MITRE), in collaboration with and on behalf of CDC. A two-step approach was used to gather input from a diverse group of organizations representing CBOs that support PEH. First, an environmental scan was performed, which included key informant interviews (KIIs) and an electronic survey. Second, a technical expert panel (TEP) was convened to review the results of the environmental scan and suggest key recommendations.

### Environmental scan

The purpose of the environmental scan was to obtain primary information about the various barriers and facilitators that CBOs face in data collection and sharing. Semi-structured key informant interviews (KII) were the primary information source. Potential CBO participants were identified through lists of Continuum of Care (CoC) and subject matter expert input. A CoC is a regional or local planning group that coordinates housing and services funding for families and individuals experiencing homelessness. A primary focus was placed on CBOs who operate a range of service deliveries, such as housing, counseling, and medical services, and those coordinating aggregated data about PEH, with a secondary focus on HUD grantees. We collected information about each CBO’s mission, community engagement, geographic location, available services, and clientele socio-demographics. Executive and director-level personnel at the stated CBOs were purposively sampled to gather a wide variety of perspectives across CBOs representing diverse localities, service types, and clientele characteristics. In some cases, these leaders extended the interview invite to their trusted data policy staff and others with knowledge of requirements levied on their CBO by funding suppliers, who could provide further context into the process and mechanism of data collection, storage, and collection for their organization. A semi-structured interview guide was used and all interviews were conducted exclusively by MITRE staff and recorded with the permission of each interviewee. The questions in the interview guide included the populations served, methods for collecting and sharing data, challenges during data collection and sharing, and possible solutions to overcome challenges.

Transcripts were then imported into NVivo software, and thematic taxonomy used to code and analyze each transcript based on six project objectives: (1) Understand factors that motivate CBOs to improve data quality and sharing; (2) Review current data collection processes; (3) Ascertain essential data elements collected and gaps; (4) Understand current data sharing activities between public health agencies and CBOs; (5) Understand the needs related to data sharing requirements to maintain funding to support organization’s mission; and (6) Identify unique data sharing limitations and opportunities for specific population data sets and data elements. Each objective was assigned two qualitative coders who were responsible for querying all transcripts for relevant statements and performing a thematic analysis.

Additionally, to expand the representativeness of data, a survey was developed based on the major themes that emerged from the qualitative data and was distributed to 992 CBOs (Appendix 1). This quantitative survey assessed for the degree of agreement with KII findings among a larger group of CBOs.

### Technical expert panel

The TEP was established to gather additional in-depth expert opinion and proposed solutions to the data-related challenges identified in the environmental scan, and to use this feedback to support the development of strategic recommendations. We identified potential TEP members and member organizations through stakeholder engagement during KIIs and through subject matter experts.

The 20 members of the TEP were representatives from 16 organizations, including individuals from federal and state agencies, interagency councils, national and local non-governmental organizations, healthcare providers, and community programs that provided information from the field through three half-day sessions over the course of two months. Members of the TEP were selected to represent a variety of service delivery types, regions of service, and areas across homeless health, social services, healthcare, and housing. We selected two co-chairs from national CBOs who had professional experience both leading collaborative initiatives across diverse agencies and with connecting health data to homelessness data. TEP members reviewed the findings from the environmental scan and provided recommendations to reduce identified gaps in CBO data collection and sharing efforts. In a series of three interactive sessions, we invited TEP members to challenge, validate, or augment the initial environmental scan findings and to validate and prioritize identified solution areas.

### Ethical considerations

The Centers for Disease Control & Prevention Institutional Review Board (FWA00001413) considered this study not human subjects research because it was conducted as part of public health surveillance (45 CFR 46 § 46.102).^§^ Both ethical approval and informed consent were hence waived as per the The Centers for Disease Control & Prevention Institutional Review Board. Where applicable, all data were anonymized and aggregated by respondent type prior to sharing with CDC staff. CDC staff were not present during KII data collection and were non-speaking observers during TEP sessions.

## Results

### Environmental scan

We conducted 20 KIIs with individuals or small groups from 19 CBOs across 16 states. Additionally, we received 125 electronic survey responses (response rate = 12.6%). (Supplemental Table 1).

The environmental scan findings were categorized into three themes: data collection, infrastructure, and data sharing & partnerships. Additionally, the environmental scan included the identification of key partners who provide vital support to people experiencing homelessness and those who work with the population (Supplemental Table 2).

### Data collection

CBOs align data collection with funder reporting requirements and use multiple interfaces to collect and enter data. CBOs reported that this fragmentation resulted in decreased data accuracy, timeliness, and data comparability between CBOs. Although universal data elements are required in HMIS, a data system used by 96% of CBO survey respondents, other funding sources often required use of custom systems, limiting the ability to combine data. CBOs reported a lack of designated funding for data management, staffing, and technology, limiting the feasibility of future improvements. CBOs also reported that obtaining consent to collect data from PEH was not always possible, which limited collection of demographic information and other privacy-protected data.

### Infrastructure

CBOs reported that annual HUD reporting changes required them to make changes to their systems and processes. With each shift in requirements, CBOs reported spending resources and staff time to develop and implement trainings. Insufficient staffing and staff turnover results in untrained, undertrained or overburdened staff and contribute to data gaps and low data quality.

CBOs also reported that outdated or incompatible technology was costly to upgrade. The inability to upgrade to integrated systems results in increased manual data entry, delays in entering data once collected, and duplication of data entry into siloed systems; these factors divert staff time from service provision and introduce increased opportunities for human error. CBOs reported that shared use of the same IT system, tool, or funding source could facilitate more sharing among different CBOs. Specifically, a unified system, set of tools and associated requirements would help to standardize system and data element requirements.

### Data sharing & partnerships

CBOs with different areas of focus did not always have the mechanisms to share data between organizations. CBO interviewees felt that data sharing would benefit their ability to provide needed services to specific populations and that increased sharing of certain data elements, such as youth status, substance use, behavioral health issues, and domestic violence history would be particularly helpful provided the necessary agreements and privacy protections were in place. This information was collected by some CBOs but not shared laterally, resulting in data systems that were siloed by category. CBOs frequently suggested that improved data-sharing between local CBOs would be an opportunity to decrease redundancy and increase quality of services to individual clients. In contrast, sharing data upwards to the jurisdictional or federal levels with minimal feedback was perceived as less useful. They further expressed that there were opportunities for external partnerships to grant limited HMIS access to other CBOs or public health agencies, particularly for individual case collaboration across jurisdictions. While no specific regional initiatives were mentioned, discussions did touch on the potential for broader data sharing through state data warehouses, which could support cross-collaboration efforts.

### Technical expert panel

TEP members endorsed and reaffirmed the findings of the environmental scan, while increasing the depth of content and context. In consolidating the TEP’s expert opinions, experiences, and individual recommendations, much of the TEP discussion emphasized the need to increase national coordination through a federal interagency working group to support cohesive data modernization efforts on housing and health. Specific suggested activities that would be facilitated by this inter-agency collaboration were consolidated under two objectives.

#### Strategic objective 1: increase health and housing data interoperability through stronger interagency and cross-sector collaboration

TEP members confirmed that fragmented and poorly defined homelessness data was a central problem and expressed that a long-term and collaborative vision was needed for improved data. TEP members rated system interoperability activities as high potential impact, including interagency coordination around data standards and requirements.

To address the issue of siloed data, TEP members suggested that a longitudinal effort was needed to improve integration of health and housing data, first across multiple federal agencies^1^, and then between the sectors of CBOs, healthcare, and public health. TEP members highlighted two key initial steps. First, a validated and standardized instrument to capture housing and homelessness status was identified as a critical need, specifically a set of questions where responses would be classified in a standardized way that aligns to the same federal definition of homelessness, similar to the instrument that is currently under validation at CDC. Second, development and adoption of housing status data standards are needed (e.g., in U.S. Core Data for Interoperability standards used for health information exchange). At the same time, coordination between federal grantors could streamline and consolidate data submission requests. After these initial steps, multi-sector system interoperability could become possible, including regular data sharing between CBOs, healthcare organizations, and public health. However, TEP members acknowledged that this level of data coordination would require discussion between federal stakeholders and multi-sector partners to create a cohesive plan for data interoperability. A consensus at the federal level would empower CBOs to more confidently move forward with data-related plans and investments. With improved data sharing and data standards, eventually there could be integrated analyses to provide a better real-time understanding of how to meet PEH needs.

Shorter-term data sharing activities were also endorsed. TEP members stated that increased visibility of how federal agencies were using data provided by CBOs could improve engagement in data sharing. This could be further enhanced by periodic reporting of aggregated data back to CBOs from grantors and public health agencies at the federal and state, tribal, local and territorial (STLT) levels. In addition to enhanced data feedback, some TEP members stated that data warehouses at a jurisdictional level had been a useful resource, bringing together data from multiple data sources, including systems containing housing data and health data.

#### Strategic objective 2: enhance technical assistance, tools, and other resources to support improved CBO data collection

TEP members noted that federal agencies could streamline available technical assistance and tools for data collection and sharing. First, they suggested that a directory of available technical and training resources would be highly useful. Desired topics were diverse and included data integration, culturally competent and trauma-informed practices for data collection and communication, and management of finances and human resources. A peer-to-peer component to the directory could highlight already-developed materials shared by CBOs; this could reduce duplication of efforts by providing templates of frequently needed documents (e.g., data use agreements) or a centralized source of shared training materials adaptable to local settings. TEP members also suggested that a streamlined pathway to request technical assistance from relevant federal agencies would be appreciated; inquiries could be used to identify topics for future resources or flag areas of confusion in need of increased interagency coordination.

Second, TEP members identified value in learning collaboratives, which are forums where CBOs share best practices in educational approaches, leadership structure, and logistical management with each other. Networks of learning collaboratives could serve as fora that are inclusive of voices from the field, as well as opportunities for federal engagement. Their collective perspectives could inform and complement long-term efforts on data standards and interoperability.

#### Activities to support the strategic objectives

To outline potential action items, MITRE compiled thirteen activities suggested by the TEP. Each activity corresponded to one of the strategic objectives above and was endorsed by TEP members as an action item that could contribute towards improving the collection, exchange, and infrastructure for CBO data on homelessness and health (Table 1; Supplemental Table 3). The suggested federal activities build on and aim to improve and integrate existing cross-agency initiatives.

## Discussion

We identified the highest-priority issues of CBOs serving PEH surrounding data collection systems, infrastructure, data sharing, and data partnerships through an environmental scan and the subsequent convening of a panel of technical experts. While discrete issues such as manual data entry, siloed organizations, and staffing issues were important challenges to data quality, the highest priority recommendation of the TEP addressed was the need for collaboration on increased system interoperability with defined data elements, and the tools and resources to implement these best practices. TEP recommendations provided important, field-endorsed perspectives to support CBOs who work closely with PEH to improve of data collection and coordinate data system modernization.

The TEP approach gathered perspectives for strategies and solutions to address common CBO data challenges from organizations that are working directly with PEH. This approach can avoid potential blind spots or misguided assumptions that could occur when solutions and projects are generated without formative input from the field level. TEPs are often used to gather the diverse perspectives of individuals with unique expertise and direct personal experience, and their informed discussions can provide new insights into specialized topics [10]. The format can also be an efficient way to engage specialized professionals; in this case we were able to gather expert information from the field through three half-day sessions over the course of two months. TEPs are a common information-gathering method among different fields of research, and have recently been engaged by the National Institutes of Health for data standardization, patient-centered clinical decision support, and prehospital pain management guidelines [11–13]. Also, the qualitative findings from the environmental scan, coupled with the critical review and prioritization of the TEP, filled a unique need by supporting and documenting partner-generated solutions and strategies to help improve data collection and sharing for PEH.

While there are limited publications on this topic, the issues and solutions identified in KIIs and endorsed by the TEP are supported by available literature on the COVID-19 pandemic. During this public health emergency, CBOs were seen as trusted messengers among PEH [14]. However, in alignment with our findings, some CBOs may feel unprepared to collect and share data. One study found that research training for CBOs with mentoring, specific curriculum, and technical support could change the way CBOs contemplate data and research [15]. Another study found that there is an increased need from CBOs to provide basic services during public health emergencies, and issues arise, such as organization challenges, staff burnout, and issues with emergency/disaster management, which could hinder data collection and sharing if proper training is not in place before public health emergencies occur [6]. In addition, the central concern around fragmented and poorly standardized data was consistent with a study examining data from 64 US jurisdictional health departments, which found that COVID-19 incidence rates could not be validly compared between jurisdictions due to differences in definitions of homelessness and systems used to verify homelessness [9]. CDC and other federal entities can use the lessons learned from the COVID-19 pandemic response to improve future public health emergency response and data collection [16]. For example, efforts to standardize housing information collected across health, behavioral health, and social service providers, regardless of funding source, and public health, while ensuring alignment with both value sets for a future standardized question set and the HMIS minimum reporting categories for housing status and homelessness, would improve data comparability and support more effective service coordination across multiple sectors, including health, housing, public health, and social services.

### Limitations

This project is subject to limitations. While key informants were selected to represent a variety of service delivery types, geographic regions, sectors, and size of operation, KII findings may not include all CBO experiences. We attempted to address this issue by also sending a secondary survey to a large number of CBOs nationally. Additionally, although TEPs gather the perspectives and experiences of uniquely qualified individuals, these individuals are not representative of all CBOs. TEPs are not intended to constitute a formal data collection process or determine current or future federal activities. Finally, suggestions for potential approaches were generated by the TEP without knowledge of federal, state, and local capacities and constraints, which may impact feasibility.

## Conclusion

CBOs are trusted providers of a variety of resources to PEH, and they can play a unique role in collecting homelessness data to enhance the efforts of public health to address the needs of this important population. This work gathered field-endorsed options to enhance the quality of data collected by CBOs and improve the ease of sharing with public health systems. Through increased collaboration at all levels and coordinated action, improved data to better support the health of PEH is an important and achievable goal.

**Table 1:** Suggested federal activities to improve the collection, exchange, and infrastructure for data on Homelessness and Health *

Strategic Objective 1 (SO1): Support Community-Based Organizations’ data collection by providing technical assistance and resources.
Support Data Warehouses
Study the utility and cost-effectiveness of state data warehouses to improve data collection and evidence-based interventions.
Provide Technical Assistance & Guidance
Develop standard technical guidance and assistance to assist CBOs with the collection of accurate data.
Create turn-key templates, tools, and guidelines needed by CBOs to enhance data activities (e.g., data sharing agreements).
Assist with the creation of standards around the ethical collection of behavioral and mental health related data.
Provide trainings on HMIS and data quality improvement.
**Strategic Objective 2 (SO2)**: Enhance housing and health interoperability by strengthening interagency partnerships and collaboration.
Establishing Common Definitions
Develop a common data dictionary (standardized definitions across state, federal, and local agencies) and a common data model
Provide Best Practices for Data Reporting
Encourage regular data feedback from public agencies to community-based organizations.
Streamline reporting to Federal agencies by mapping and de-duplicating data submission requests across multiple grantors
Foster Data Sharing Among Partners
Provide tools, templates, and resources (e.g., plain language guidance on relevant legal principles) to assist CBOs with data sharing.
Develop a validated and standardized instrument to capture housing and homelessness status.
Promote interagency technology and training efforts related to health and housing data
Coordinate CBO Learning Collaborative Networks
Facilitate CBO sharing of best practices for data and data-sharing
Utilize learning collaboratives to gather CBO perspectives on data issues (e.g., feedback on data elements within reporting requirements, development of use cases)

* Solutions proposed by the Technical Expert Panel (TEP) that represented approaches to issues that fall outside of the scope of federal public health for direct action are included in Supplemental Table 3

## Electronic supplementary material

Below is the link to the electronic supplementary material.

Supplementary Material 1

Supplementary Material 2

## Data Availability

The data that support the findings of this study are available on request from the corresponding author.

## Abbreviations

CBO: Community-based organization
PEH: People experiencing homelessness
KII: Key informant interviews
TEP: Technical Expert Panel
HMIS: Homeless Management Information System

## Footnotes

1 Federal agencies who may have health or housing related data identified by TEP members included U.S Department of Housing and Urban Development (HUD), Substance Abuse and Mental Health Services Administration (SAMHSA), Department of Education, Department of Justice, and Department of Labor; this list may not be exhaustive.
