# Supplementary material for: Improving data on homelessness and health: partnering with community-based organizations
Source: BMC Public Health. 2025 Aug 26;25:2918. doi: 10.1186/s12889-024-20954-3 (PMC12379530; doi:10.1186/s12889-024-20954-3)
Supplement: Supplementary file 1 — Supplementary Material 1 [file 12889_2024_20954_MOESM1_ESM.docx]

**Appendix 1: Electronic Survey**
