# Supplementary material for: Improving data on homelessness and health: partnering with community-based organizations
Source: BMC Public Health. 2025 Aug 26;25:2918. doi: 10.1186/s12889-024-20954-3 (PMC12379530; doi:10.1186/s12889-024-20954-3)
Supplement: Supplementary file 2 — Supplementary Material 2 [file 12889_2024_20954_MOESM2_ESM.docx]

| **Supplemental Table 1. Quantitative Survey Responses from 992 Community Based Organizations** | | | |
| --- | --- | --- | --- |
| **Question** | **Response** | **Percent Yes** | **Total Responses** |
| Which of the following describes your organization's barriers to capturing data about its programs and/or direct services? | Poor data quality | 46.60% | 103 |
|  | The inability to operate between systems | 45.63% |  |
|  | Other | 33.01% |  |
|  | Inadequate software capabilities | 16.51% |  |
|  | Limited data access | 15.53% |  |
| Which of the following actions does your organization conduct on behalf of its programs and/or direct services? | Tracking program metrics | 92.59% | 108 |
|  | Auditing data regularly | 88.89% |  |
|  | Entering data on behalf of programs | 48.15% |  |
| Which of the following statements are true about capturing client data at your organization? | We have a well-defined intake process | 74.19% | 62 |
|  | We have frequent data audits that allow for continuous quality improvement | 66.13% |  |
|  | We use electronic (e.g., smartphone) applications in the field to support the data collection process | 43.55% |  |
|  | We have adequate funding for data capture and management | 37.10% |  |
|  | We have easy-to-use data systems | 37.10% |  |
|  | We have adequate staffing | 35.48% |  |
|  | Other | 4.84% |  |
| With which partner(s) does your organization currently share data? | Providers within the Continuum of Care | 87.39% | 111 |
|  | Continuum of Care lead agency | 85.59% |  |
|  | State government | 50.45% |  |
|  | Local health department | 20.72% |  |
|  | Other | 23.42% |  |
|  | Medical providers | 18.92% |  |
|  | School system | 9.91% |  |
|  | My organization currently does not share data | 1.80% |  |
| How does your organization currently share data with the other organization(s)? | Batch file sharing (e.g., CSV, database export, etc.) | 59.62% | 104 |
|  | Manual data entry | 34.62% |  |
|  | Other | 33.65% |  |
|  | Direct system-to-system data transfer | 24.04% |  |
| My organization has access to health data about its client population |  | 28.83% | 111 |
| Which of the following platforms or systems does your organization use? | HMIS specific systems | 96.36% | 110 |
|  | Internal systems (e.g., Excel, databases, etc.) | 75.46% |  |
|  | Federal reporting systems | 51.82% |  |
|  | State data warehouses | 29.09% |  |
|  | Local or state public health platforms or systems | 12.73% |  |
|  | Other | 12.73% |  |
|  | Health provider platforms or systems | 8.18% |  |
| Which of the following are the top 5 most important for your organization to know about its clients? | Duration of client's homelessness | 95.41% | 109 |
|  | Disability status | 80.73% |  |
|  | Veteran status | 68.81% |  |
|  | Age or youth status | 66.06% |  |
|  | Race/ethnicity or preferred language | 60.55% |  |
|  | Chronic health conditions | 58.72% |  |
|  | Substance use or abuse history | 39.45% |  |
|  | Sexual orientation and gender identity | 16.51% |  |
|  | Other | 13.76% |  |
| Please use this space to share or expand upon anything that you feel would be helpful for us to know in order to understand your organization's situation or context and your data management practices or challenges |  | 26.61% | 109 |
| What change(s) would be most helpful to improve data practices for organizations like yours that serve people experiencing homelessness? |  | 55.05% | 109 |

| **Supplemental Table 2: Key Partners for collaboration in activities to improve CBO data quality and sharing** | | |
| --- | --- | --- |
| **Organization** | **Relationship to CBOs** | **Local, State, or National** |
| Community Based Organizations (CBO) | Inclusive of public HUD funded and private non-HUD funded organizations. | Local |
| People Experiencing Homelessness | Clients served by CBOs. | Local |
| Continuum of Care (CoC) | A local collection of CBOs that coordinate services throughout the CoC area (county, region, state). The CoC lead is typically the data lead and responsible for reporting to funders. | Local |
| Healthcare Providers | Providers assist people experiencing homelessness with any medical needs such as substance use, dentistry, vaccinations. These services are either provided directly or are coordinated through the CBO. | Local |
| Mental Health Providers | Therapy and behavior health services are provided to people experiencing homelessness, especially to those that have a mental disorder or past trauma. These services are either provided directly or are coordinated through the CBO. | Local |
| Health Information Exchange (HIE) | Some regions share information across hospitals using HIEs that CBOs can access as a data source for new people experiencing homelessness entering their care. | Local |
| School Districts | Families experiencing homelessness are still required to send kids to school as part of their care. School districts may also identify possible people experiencing homelessness through interactions with the kids for CBOs to follow up with. | Local |
| Local Justice Department | Some CBOs provide legal services or are supporting people experiencing homelessness with criminal records that requires information sharing. CBOs also provide services to people experiencing homelessness transitioning from prison or found from law enforcement that requires coordination. | Local |
| Homeless Management Information System (HMIS) vendors | HMIS systems are the main data repository for CBOs and is used for reporting to funders. In order to be classified as an HMIS system, HUD has a set of requirements that vendors must meet in their system. The requirements change yearly, which limits how many HMIS vendors CBOs can choose from. | Local |
| Public Health Agencies (PHA) | CBOs are required to report disease surveillance and other pandemic related data (for COVID-19) to their local PHA. There are also special programs related to people experiencing homelessness with HIV/AIDs that need to be reported on. | Local/State |
| Local Government | CBO must report data to either the city or state government department in charge of managing people experiencing homelessness for their region. Policies and mandates are also handed down to CBOs that they must adhere to. | Local/State |
| Department of Housing and Urban Development | HUD is the federal agency in charge of managing people experiencing homelessness and gives most of the funding that CBOs use to provide services. CBOs are required to report Universal Data Elements and program specific data elements up to HUD regularly as a condition of the funding. | National |
| HUD’s Special Needs Assistance Programs (SNAPS) | SNAPS is the office within HUD that CBOs interact with as part of their grant funding. | National |
| Centers for Disease Control and Prevention | Provides guidance to PHAs on how to conduct disease surveillance and surveys on people experiencing homelessness, especially during public health emergencies like COVID-19, to help identify gaps in public health data available for PEH. | National |
| Department of Education | Utilizes the local school districts to collect data on people experiencing homelessness students independently from CBOs, which causes a duplication of data collection. | National |
| Department of Justice | Provides guidance to local justice departments and law enforcement on how to support people experiencing homelessness. This support requires collecting information on the people experiencing homelessness that is not always shared with CBOs. | National |
| Department of Veterans Affairs | Any people experiencing homelessness veterans qualify for additional programs funding through Veterans Affairs. These programs have additional reporting requirements specific to their service history. | National |
| The Administration for Children and Family (ACF) | Families or youth experiencing homelessness that have recently left the foster program can utilize additional support programs through ACF. These programs have additional data that needs to be reporting to ACF. | National |
| National Homelessness organizations (e.g., National Health Care for the Homeless Council) | Large organizations that work across the nation to support CBOs with training, research, and advocacy (e.g., lobbying government for new laws/policies to assist CBOs). | National |
| Health Resources and Services Administration (HRSA) | The HIV/AIDS Bureau of HRSA administers the Ryan White HIV/AIDS Program, which provides a comprehensive system of care for people with HIV that CBOs can utilize for their people experiencing homelessness. | National |

| **Supplemental Table 3: Proposed solutions representing valid approaches to existing issues but fall outside of CDC’s scope for direct action*** |
| --- |
| Provision of funds to support non-HUD funded CBOs. |
| Invest in interoperable systems (e.g., HMIS, electronic health record, housing database) with rigorous privacy considerations. |
| Increase information technology-focused grant funding for data modernization. |
| Identify a unified system provider that reduces friction in training and operation. |
| Define a threshold level of compatibility or interoperability between mandated reporting systems and CBO’s existing infrastructure. |
| Provide and/or facilitate quality hardware and software to grantees. |
| Provide additional funds to CBOs equal to administrative costs for data collection, or study cost-effectiveness of utilizing such funds. |
| *These approaches are provided to bring create discussion and consideration between CDC and their partners whose scope of work better address these issues, as CDC cannot address these issues without partners’ support. |
